# Intravenous Thrombolysis in Ischemic Stroke Patients with Intracranial Neoplasms: Two Cases and a Literature Review

**DOI:** 10.1155/2011/503758

**Published:** 2011-10-05

**Authors:** William Neil, Bruce Ovbiagele

**Affiliations:** Stroke Center and Department of Neurosciences, University of California San Diego, Medical Offices North, 200 West Arbor Drive, San Diego, CA 92103, USA

## Abstract

Based on exclusion criteria in the landmark NINDS-rtPA trial, current expert consensus guidelines preclude the use of intravenous recombinant tissue plasminogen activator (IV rtPA) in acute ischemic stroke (AIS) patients with intracranial neoplasm. There are only 3 published cases of administration of IV rtPA to AIS patients with intracranial neoplasms in the literature. Two of these published cases involved malignant brain parenchymal lesions discovered only after rtPA was inadvertently given, and one of these cases was associated with hemorrhage within the tumor. In this paper, we report two cases of administration of IV rtPA in AIS patients with intracranial neoplasms observed on neuroimaging prior to IV rtPA administration. In both cases, the tumor was outside of the brain parenchyma. The first case was an acoustic schwannoma and the second a falcine meningioma. Neither case was associated with intratumoral hemorrhage as of at least one week following IV rtPA treatment. More published cases are definitely warranted, but our experience with these two cases suggests that administration of IV rtPA to AIS patients in the presence of extraparenchymal brain tumors may not necessarily precipitate intra-tumoral bleeding and thereby worsen clinical outcomes.

## 1. Introduction

Fifteen years after Food and Drug Administration approval of intravenous recombinant tissue plasminogen activator (IV rtPA) for treatment of acute ischemic stroke (AIS), only a substantial minority of AIS patients (<2%) of strokes receive this proven therapy [1]. While several barriers exist to increasing utilization of rtPA among AIS patients [1], there are many patients who do not receive this efficacious treatment on the basis of product labeling derived from the landmark NINDS-rtPA trial exclusion criteria [2]. For the most part, the selection of NINDS-rtPA exclusion was based on theoretical concerns and it is increasingly being shown that utilizing rtPA in patients with some of these criteria may not necessarily result in unfavorable outcomes [3]. One of these exclusion criteria, presence of intracranial neoplasm, is considered a contraindication to IV rtPA use AIS in because of increased intracranial bleeding risk [4]. However, the actual risk of systemic thrombolysis-precipitated intracranial bleeding in AIS patients is unknown, and as far as we are aware, there are only 3 cases of rtPA use in AIS patients with intracranial neoplasm in the literature [5–7]. We report our experience with the administration of IV rtPA to two AIS patients with intracranial tumors and review the literature.

## 2. Case 1

**Figure 1:**
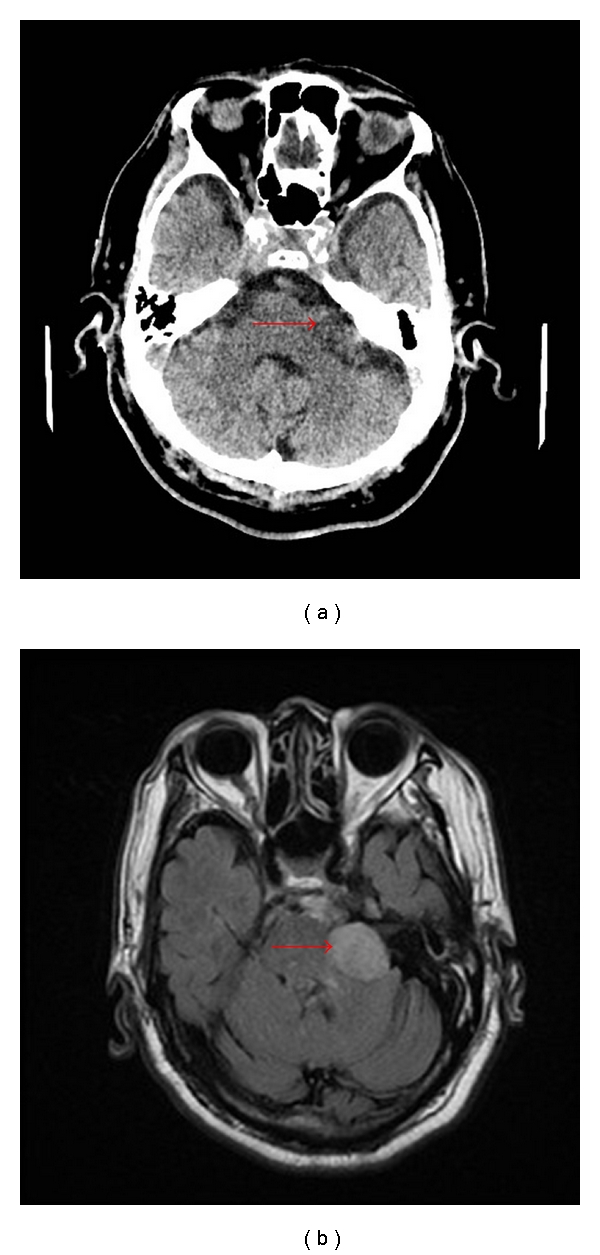
(a) Non-contrast head CT showing a heterogeneous lobulated mass within the left cerebellopontine angle (arrow). (b) Followup brain MRI in same patient 3 days later.

A 77-year-old man developed sudden onset left upper, and lower extremity weakness. His past medical history included hypertension, hyperlipidemia and type 2 diabetes. He had experienced two prior strokes (the more recent one two years prior to this presentation) which resulted in residual mild left hemiparesis. On admission, blood pressure was 146/50 mmHg, and pulse was 56 beats per minute and regular. Neurological examination revealed flaccid left lower extremity weakness affecting proximal and distal muscles. He was unable to walk. National Institutes of Health Stroke Scale Score (NIHSSS) was 4. Noncontrast head CT demonstrated a hyperdense right anterior cerebral artery (ACA) and a heterogeneous lobulated mass within the left cerebellopontine angle measuring 3.3 × 1.3 cm. There was mild mass effect on the adjacent pons (Figure 1(a)). CT angiogram showed an abrupt cut off of the right ACA, and CT perfusion showed a defect in the corresponding ACA territory. He was given IV rtPA 5 mg IV bolus followed by 44.5 mg IV, based on weight. Initially, his deficits improved, but then returned eight hours later with greater intensity. Brain MRI performed 3 days later did not demonstrate bleeding either in the cerebellopontine angle mass or elsewhere (Figure 1(b)). He was eventually discharged to an acute rehabilitation facility. Three months later, he had mild residual weakness of the left leg, and he was again able to walk with a cane.

## 3. Case 2

**Figure 2:**
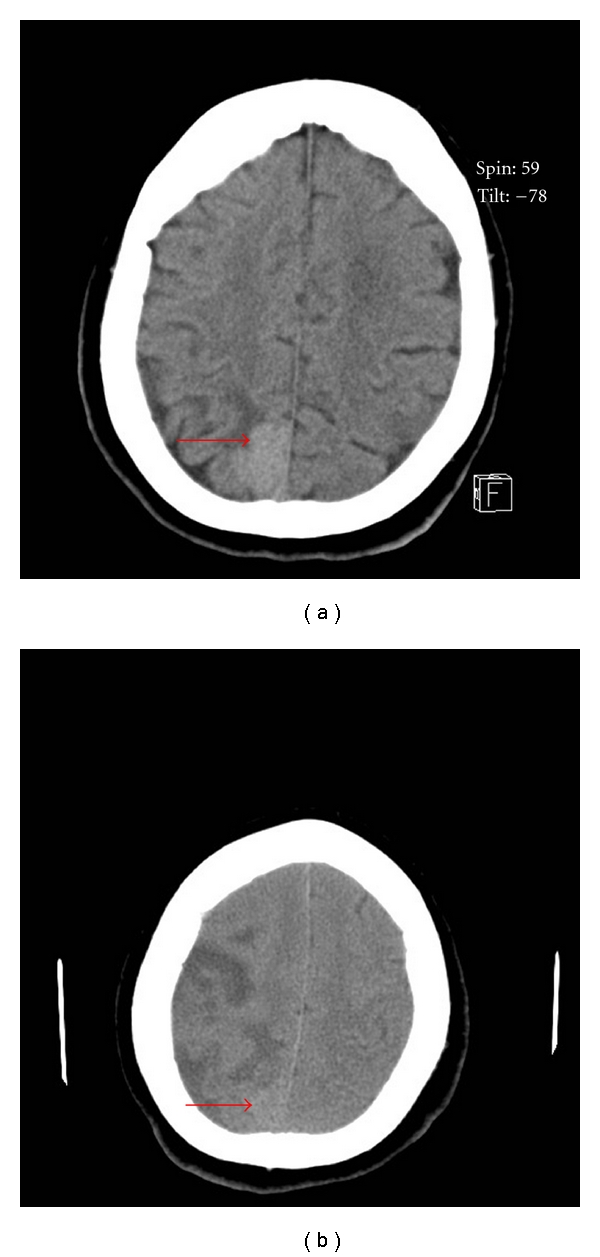
(a) Initial head CT showing parafalcine mass (arrow). (b) Head CT 36 hours after t-PA.

A 74-year-old woman arrived after a fall witnessed by family members. Past history included type 2 diabetes, hypertension, and hyperlipidemia. Initial blood pressure 165/77 mmHg, and pulse was 83 beats per minute and irregularly irregular. Neurological evaluation revealed right gaze deviation, flattening of the left nasolabial fold, and dysarthria, flaccid left arm with no strength, weakness of left leg (2/5 strength), and left-sided neglect. NIHSSS was 13. Noncontrast head CT demonstrated a hyperdense right middle cerebral artery, bilateral basal ganglia calcification, and an occipital falcine mass (Figure 2(a)). She received 62 mg of IV rtPA based on her weight 90 minutes after stroke onset. Shortly after completion of rtPA infusion, the patient became more lethargic and complained of a worsening headache. She was intubated for airway protection due to her decreased level of consciousness and diminished gag reflex. A repeat head CT at the time showed no evidence of hemorrhage. At 48 hours after treatment, her NIHSS was 15 and she was able to follow one step commands on her right side, but still had profound neglect and flaccid arm weakness. She was still able to move right leg within the plane of gravity. Head CT performed at 36 hours demonstrated increasing edema in the distribution of the large right MCA infarct, with midline shift measuring 5 mm, but there was no evidence of hemorrhagic transformation or intratumor bleeding (Figure 2(b)).

## 4. Discussion

We report two cases of safe administration of IV t-PA in acute ischemic stroke patients with intracranial neoplasm. In both cases, the tumor was outside of the brain parenchyma. The first case was possibly an acoustic schwannoma, and the second a falcine meningioma. Neither case was associated with intratumor hemorrhage. 

As already noted, there are extremely few cases of IV t-PA use in AIS patients with intracranial neoplasms reported in the literature. Two of these cases involved initially undetected malignant intracranial neoplasms, while the remaining one involved a benign intracranial neoplasm seen prior to treatment. For instance, a 57-year-old man with a left temporal lobe glioblastoma multiforme (GBM) (subsequently diagnosed after magnetic resonance imaging and neurosurgery days later) was inadvertently given IV rtPA after a normal initial head CT. There was no evidence of blood by that MRI or follow-up head CT. The patient became asymptomatic on the seventh day [5]. However, in another case of malignant brain neoplasm, an 80-year-old man, also inadvertently given IV rtPA for a presumed AIS, worsened twenty hours after receiving rtPA, and CT revealed a left temporoparietal hemorrhage. Follow-up MRI 2 months later demonstrated a GBM in the hemorrhage bed [6]. More similar to the two cases we have presented in this paper, that is, presumed benign intracranial neoplasms observed on neuroimaging prior to IV rtPA, therapy, is the case of a 60-year-old woman, who presented with AIS and was noted on head CT to have a 2 cm frontal meningioma. After receiving IV rtPA the neurological deficits improved. Brain magnetic resonance imaging, 3 days after onset, showed an acute infarction at the right basal ganglion without hemorrhage and no bleeding within the meningioma. Modified Rankin Scale was 1 at 1-month and 0 at 6-month follow-up [7]. 

Admittedly, the number of reported cases of rtPA treatment for AIS in patients with intracranial neoplasms is far too small to make any definitive inferences, but there is a suggestion, based on previous cases as well as our current report, that the presence of a presumably benign intracranial neoplasm seen on neuroimaging prior to IV rtPA treatment may not necessarily contribute to unfavorable imaging or clinical outcomes in AIS and perhaps should not preclude treatment. Further supportive evidence of possibly little harm from using intravenous thrombolysis among vascular disease patients with benign intracranial neoplasm may be seen among the few reported cases of i.v. rtPA use in nonstroke patients [8, 9]. Three patients with acute myocardial infarction and benign intracranial neoplasms have been reported in the literature, two of these patients had coexisting meningiomas, while one of them had a pituitary adenoma [8, 9]. Posttreatment head CT scans did not show any evidence of intracranial or intratumor bleeding [8, 9]. The implied low risk of harm in the aforementioned cases of I.V. rtPA therapy in the context of benign intracranial neoplasm notwithstanding, more cases are needed to bolster the premise that the presence of such tumors should not deter treatment in otherwise eligible AIS patients.
